# Thigh and Shank, Kinetic and Potential Energies during Gait Swing Phase in Healthy Adults and Stroke Survivors

**DOI:** 10.3390/brainsci12081026

**Published:** 2022-08-02

**Authors:** Krisanne Litinas, Kristen L. Roenigk, Janis J. Daly

**Affiliations:** 1 Cognitive and Motor Learning Laboratory, Louis Stokes VA Medical Center, Cleveland, OH 44106, USA; klitinas@gmail.com (K.L.); kristen.roenigk@gmail.com (K.L.R.); 2 Brain Rehabilitation Research Center, Malcom Randall VA Medical Center, Gainesville, FL 32608, USA; 3 Department of Physical Therapy, College of Public Health and Health Professions, University of Florida, Gainesville, FL 32608, USA; 4 Department of Neurology, School of Medicine, Case Western Reserve University, Cleveland, OH 44016, USA

**Keywords:** gait, stroke, biomechanics, mechanical energies, kinetic energy, potential energy, energy conservation

## Abstract

Background/Problem. Given the treatment-resistant gait deficits after stroke and known elevated energy cost of gait after stroke, it is important to study the patterns of mechanical energies of the lower limb segments. There is a dearth of information regarding mechanical energies specifically for the thigh and shank across the gait cycle. Therefore, the purpose of the current work was to characterize the following: (1) relative patterns of oscillation kinetic energy (*KE*) and potential energy (*PE*) within lower limb segments and across lower limb segments in healthy adults during the swing phase at chosen and slow gait speeds; (2) *KE* and *PE* swing phase patterns and values for stroke survivors versus healthy adults walking at slow speed; and (3) *KE* and *PE* patterns during the swing phase for two different compensatory gait strategies after stroke,. Methods. This was a gait characterization study, a two-group, parallel-cohort study of fourteen stroke survivors with gait deficits, walking at <0.4 m/s and eight adults with no gait deficits. For testing, the eight healthy adults walked at their chosen speed, and then at the imposed slow speed of <0.04 m/s. We used a standard motion capture system and calculation methods to acquire, calculate, and characterize oscillation patterns of *KE* and *PE* of the limb segments (thigh and shank) across the gait cycle. Results. In healthy adults, we identified key energy conservation mechanisms inherent in the interactions of *KE* and *PE*, both within the thigh and shank segments and across those limb segments, partially explaining the low cost of energy of the normal adult chosen speed gait pattern, and the underlying mechanism affording the known minimal set of activated muscles during walking, especially during the early swing phase. In contrast, *KE* was effectively absent for both healthy adults at imposed slow walking speed and stroke survivors at their very slow chosen speed, eliminating the normal conservation of energy between *KE* and *PE* within the thigh and across the thigh and shank. Moreover, and in comparison to healthy adult slow speed, stroke survivors exhibited greater abnormalities in mechanical energies patterns, reflected in either a compensatory stepping strategy (over-flexing the hip) or circumducting strategy (stiff-legged gait, with knee extended throughout the swing phase). Conclusions and contribution to the field. Taken together, these findings support targeted training to restore normal balance control and normal activation and de-activation coordination of hip, knee, and ankle muscles, respectively (agonist/antagonist at each joint), so as to eliminate the known post-stroke abnormal co-contractions; this motor training is critical in order to release the limb to swing normally in response to mechanical energies and afford the use of conservation of *KE* and *PE* energies within the thigh and across thigh and shank.

## 1. Introduction

A number of methods and measures have been used to characterize locomotion in terms of optimizing energy cost, including oxygen consumption [1,2] and mechanical work performed [2]. It is well known that normal, chosen speed walking is controlled by the central nervous system to optimize energy cost through an alternating transfer between potential energy (*PE*; gravity-based) and kinetic energy (*KE*; forward progression) within each stride, as measured at the whole-body center of mass (COM) [3,4,5]. At normal chosen walking speed, this mechanical energy transfer process at the COM can optimize the amount of energy that is required from other sources such as muscle activations. Though this is valuable information, there is a dearth of information regarding the underlying mechanical energy mechanisms that produce the body COM mechanical energy patterns, specifically the lower limb segments during the swing phase (e.g., thigh and shank).

Given the treatment-resistant gait deficits after stroke and known elevated energy cost of gait after stroke [1], it is important to study the patterns of mechanical energies of the lower limb segments; that is, the study of the following relationships: relative oscillations of kinetic energy (*KE*) and potential energy (*PE*) within lower limb segments (e.g., thigh and shank); *KE* and *PE* energy relationships across limb segments; relationships of post-stroke gait deficits to the prior two sets of relationships; and the impact of post-stroke gait speed on *KE* and *PE* energies. A few studies have provided important partial information. For example, studies have shown that the overall body center of mass (COM) *KE* and *PE* are abnormally affected in post stroke gait deficits [6]. However, these studies focused on whole-body COM, and did not provide the more detailed information on the mechanical energies (*KE* and *PE*) of the limb segments that produced the overall body COM mechanical energy fluctuations. Specific information regarding gait *KE* and *PE* of the limb segments has been studied in Parkinson’s disease [7]; but in stroke survivors, there has been little study of the specific *KE* and *PE* oscillations within the lower limb segments, energy transfer within the segments, and energy transfer between the segments. In one important study, another group calculated work produced by the lower limb segments after stroke [8]. They estimated energy conservation using a pendular transduction framework; they theorized that one possible energy conservation method was using the paretic lower limb in a ‘stiff-legged’ gait [9] and the non-paretic limb compensating by producing abnormally elevated work levels to move the body forward [8]. Though not likely an effective energy conservation strategy, clinical observation and kinematic gait analysis in stroke survivors does support the use of a circumducted gait compensatory strategy [10,11,12]. However, a gap in the literature still remains on behalf of stroke survivors with persistent gait deficits, in terms of detailing the specific *PE* and *KE* relationships within each lower limb segment and across the lower limb segments, as well as interpreting these relationships in terms of specifically targeted, needed clinical interventions. Therefore, the purpose of the current work was to characterize the following: (1) relative patterns of oscillation of *KE* and *PE* within lower limb segments and across lower limb segments in healthy adults during the swing phase at chosen and slow gait speeds; (2) *KE* and *PE* swing phase patterns and values for stroke survivors versus healthy adults walking at slow speed; and (3) *KE* and *PE* patterns during the swing phase for two different compensatory gait strategies after stroke.

## 2. Methods

### 2.1. Study Design and Subjects

#### 2.1.1. Study Design

This was a cross-sectional study of each of two cohorts (stroke survivors and healthy adults), studied in parallel; for each group, we characterized mechanical gait energetics. Both descriptive analyses and statistical analyses were used to compare specific gait characteristics across gait speeds within the healthy adult group as well as some comparisons across the two groups (healthy adults vs. stroke survivors), as described in Section 2.2. below.

#### 2.1.2. Subjects

We enrolled fourteen stroke survivors (age 52–75 years; 40% female) and eight able-bodied subjects (age 50–76; 50% female) with no known neurological or musculoskeletal abnormalities. We enrolled stroke survivors who were walking at less than 0.4 m/s, which is classified as ‘household ambulatory’ [13]. Other inclusion criteria for the stroke survivor group included the following: first-ever stroke; 1–12 months post stroke; able to follow two-part commands; and swing phase gait deficits. Of the stroke survivors, 3 used a circumducted compensatory pattern (stiff-legged gait), and 11 used a steppage gait pattern (over-flexion of the hip and early flexion of the hip [12]). The study was conducted in accordance with the Declaration of Helsinki, and the protocol was approved by the Ethics Committee of the Louis Stokes VA Medical Center (Internal Review Board (IRB; Project identification code, #2008022). Subjects provided written informed consent to participate.

### 2.2. Data Acquisition and Reduction

All gait data were acquired during overground walking. Gait kinematic data were acquired at 60 Hz using the Vicon370 motion capture system (Oxford Metrics, Warwickshire, UK), a three-dimensional video data acquisition system, with seven charge-coupled device cameras configured on a 30-foot (9.14 m) walkway. Fifteen spherical reflective markers were placed at anatomical landmarks on the limbs and pelvis using a modified Hayes configuration [14]. Limb position coordinates were calculated, and three-dimensional reconstruction was performed.

Trajectories of the center of mass of the two thighs, shanks, and feet segments were calculated from the three-dimensional coordinates of respective segments using anthropometric data [15]. Data analysis and calculations were performed using custom software created for the study.

### 2.3. Calculation of Mechanical Energy

The healthy adult sample was tested at both chosen speed and at an imposed gait speed of <0.4 m/s. The stroke survivor sample was tested at their chosen speed (<0.4 m/s).

#### 2.3.1. Thigh and Shank Mechanical Energy Components

To calculate the thigh and shank mechanical energy components, the 3-dimensional center of mass trajectory data were processed by a nearly equal ripple derivative (NERD) filter [16]. The translational and rotational kinetic (*KE_s_*, *RKE_s_*) and potential (*PE_s_*) energy components were calculated for each of the lower body segments (thigh, shank, and foot) during the swing phase, as follows:

(1) $$PE_{s}=m_{s}gh_{s}$$

(2) $$KE_{s}=\frac{1}{2}m_{s}v_{s}^{2}$$

(3) $$RKE_{s}=\frac{1}{2}I_{s}\omega_{s}^{2}$$

where the subscript ‘*s*’ indicates the term ‘segment’; *m_s_* is the mass of the segment (kg), *h_s_* is the height of the segment center of mass from the floor (meters), *v_s_* is the forward velocity at the segment center of mass (m/s), *I_s_* is the moment of inertia of the segment (kg m^2^), and *ω_s_* is the angular velocity of the segment (rad/s).

Time was normalized according to percent of swing phase. All energy components were then normalized by the body mass and leg length for inter-subject comparison [17].

Total mechanical energy of a limb was calculated as the sum of the segment mechanical energy components, as follows [18,19]:

(4) $$TE=\overset{S}{\underset{i=1}{\sum }}PE\left(i,t\right)+\overset{S}{\underset{i=1}{\sum }}KE\left(i,t\right)+\overset{S}{\underset{i=1}{\sum }}RKE\left(i,t\right)$$

We focused the study on the separate thigh and shank segmental forward translational energies and did not include a detailed analysis of the rotational kinetic energy because the *RKE* component is responsible for only 10% of the total mechanical energy in gait, and even less at slow walking speeds [18]. Additionally, since the swing limb is responsible for nearly all *PE* and *TKE* thigh and shank energy changes during walking [16], we focused exclusively on the swing phase. Others [20,21] noted that the upper body ‘goes along for the ride’, and so, as an additional way to focus this study, we focused solely on the thigh and shank segments.

##### Change of Each Energy Component

We calculated the change of the energy level across the given subphase for each energy component (kinetic and potential) of the thigh and shank segments (i.e., change of the energy level across each of two specific gait subphases (Subphase 1, toe off to maximum knee flexion; and Subphase 2, from toe off to maximum hip flexion)).

(5) $$\Delta X_{Subphase\;1}=X{\left(\tau \right)}_{maximum}-X{\left(\tau \right)}_{minimum}$$

(6) $$\Delta X_{Subphase\;2}=X{\left(\rho \right)}_{maximum}-X{\left(\rho \right)}_{minimum}$$

where *X* is segment energy component (example thigh PE). *τ* and ρ, are the continuous interval from toe off to maximum knee flexion (Formula (5)) and hip flexion (Formula (6)), respectively: ΔX was assigned a negative sign if $X{\left(\tau \right)}_{maximum}$ occurred before $X{\left(\tau \right)}_{minimum}$ (thereby signaling a general decrease in amplitude across that particular subphase). Otherwise, ΔX was positive.

##### Minimum Meaningful Value of *KE*

For healthy adult slow speed and stroke survivors, we observed very small values of *KE* in thigh and shank. We arrived at an approximate minimum meaningful value of combined thigh and shank *KE* of 0.08 J/kg m by modeling the leg as a simple rigid segment and estimating the passive resistance torque at the hip torque at toe off [22]. The values of *sKE* and *tKE* for the slow speeds (stroke and control) were below this threshold; therefore, we conducted no further analysis of peaks, ranges, and latencies of these *KE* components.

### 2.4. Statistical Analysis

In SPSS, we used the Mann–Whitney two-independent-samples test for comparisons of stroke and control groups. This test was selected due to small sample size. Significance threshold was *p* < 0.05.

## 3. Results

### 3.1. Healthy Adults

#### 3.1.1. Chosen Speed

**Figure 1:**
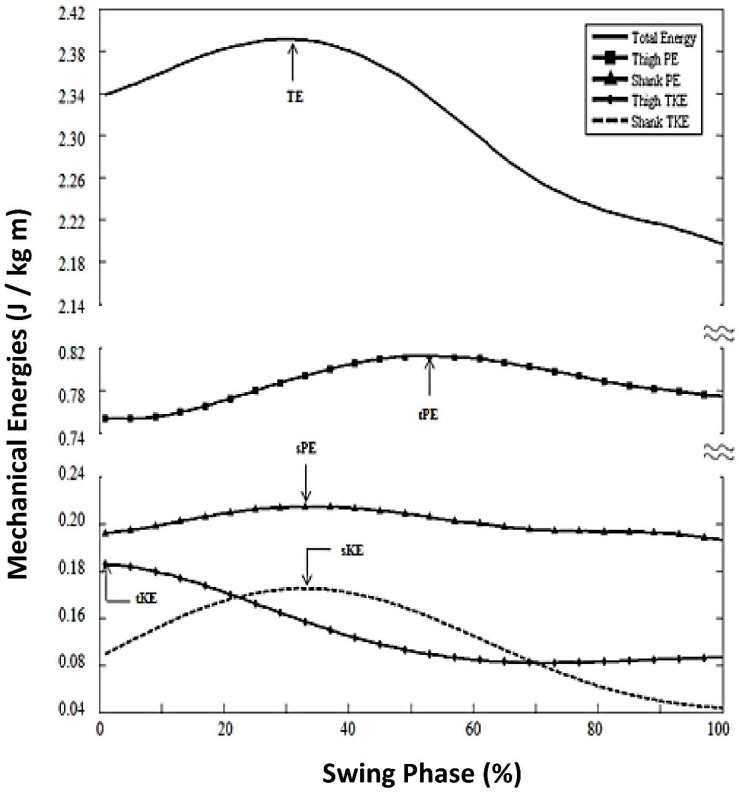
Mechanical energies for healthy adults, chosen walking speed.

**Figure 2:**
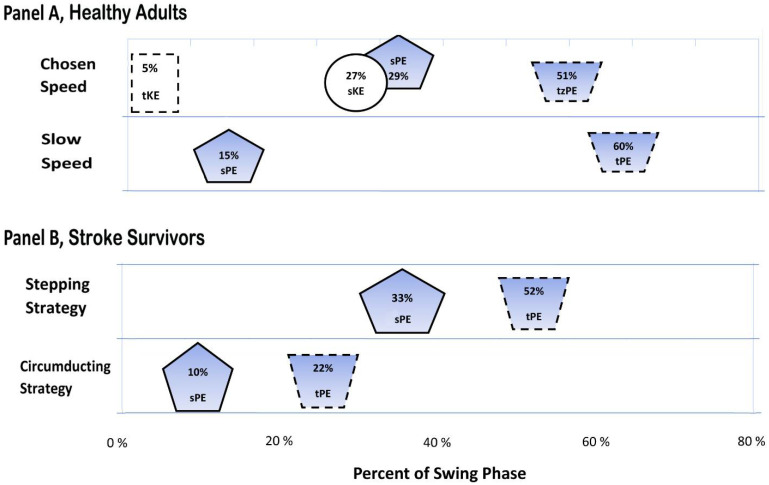
Timing of peak amplitudes of limb segment energy components for healthy adults and stroke survivors.

There were four mechanical energy characteristics of note that were present during the swing phase at chosen walking speed (mean = 1.29 m/s; range, 0.98–1.66 m/s), as follows:

(1) TE peak timing, 25% swing phase (Figure 1);
(2) Sequence of limb segment energy peaks was: 1. *tKE* (5% of swing phase); 2. *sPE* (29%) and *sKE* (27%); and 3. *tPE* (51%; Figure 2, Panel A, chosen speed). The total effect of these energy patterns was to minimize both TE slope and TE maximum oscillation as shown in Figure 1).
(3) There were specific relationships of *KE* and *PE* within each limb segment (thigh and shank; Figure 1). Thigh components (*tPE* and *tKE*) were out of phase with each other. That is, from 0% (toe off) to 35% of swing (max hip flexion), the thigh segment simultaneously decreased in *tKE* and increased in *tPE*. By contrast, shank components (*sPE* and *sKE*) were more-in-phase with one another, with both *sPE* and *sKE* peaking at 29% and 20% of swing, respectively.
(4) There were specific out-of-phase relationships of *KE* and *PE* across limb segments of thigh and shank (Figure 1). That is, from 0% to 35% of swing, thigh kinetic energy (*tKE*) decreased while shank energies (*sPE* and *sKE*) increased. Additionally, from 35% to 56%, both shank components (*sPE* and *sKE*) decreased as thigh potential energy (*tPE*) increased.

#### 3.1.2. Healthy Adults, Slow Speed (SS)

##### Similarities in Limb Segment Mechanical Energies for Slow Versus Chosen Speed, Healthy Adults

**Figure 3:**
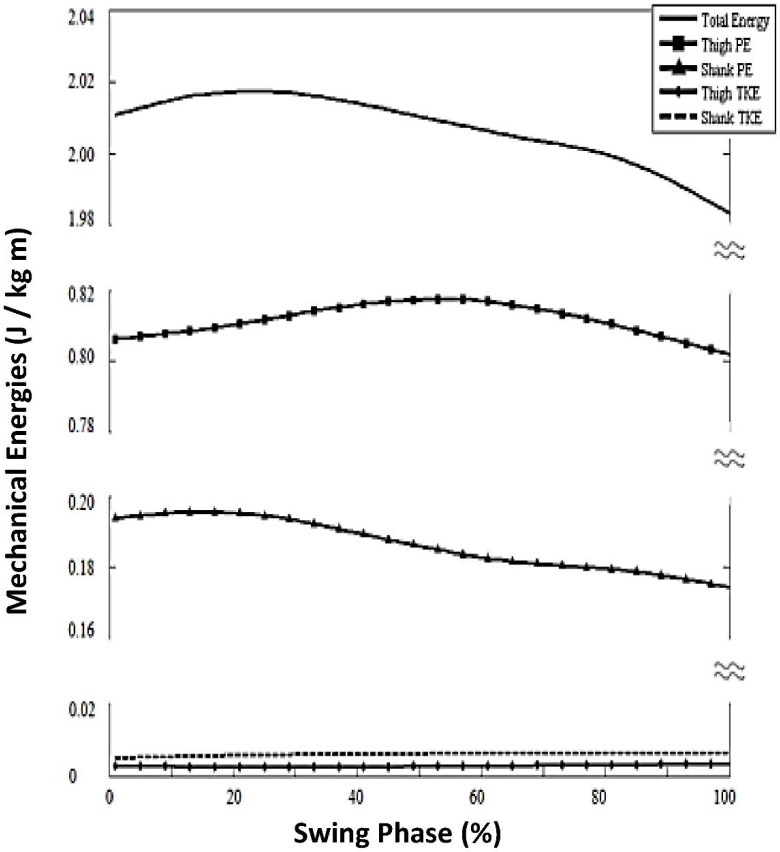
Mechanical energies for healthy adults walking at imposed slow speed.

For healthy adults at slow speed walking < 0.4 m/s (Household Ambulation Speed Classification), we found the following similarities to chosen speed walking:

(1) Overall similar TE shape and peak time (35%, chosen speed; 25% slow speed (Figure 1 and Figure 3);
(2) Sequence of *PE* energy peaks in Figure 2 (panel A, slow speed) shows *sPE* peak prior to *tPE*.
(3) Similar change (i.e., no statistically significant difference) in energy level of *sPE* range from toe off to max hip flexion (Δ*sPE_subphase2_*): For slow speed, mean = −0.011 (±0.011) J/kg m versus the chosen speed mean = 0092 (±0.019) J/kg m; *p* = 0.697).
(4) Normal out-of-phase thigh and shank relationship at mid-swing, with *sPE* decreasing while *tPE* increased (occurring from 17% to 62% of swing phase duration; Figure 3).

##### Differences in Limb Segment Mechanical Energies for Slow Versus Chosen Speed

However, there were several differences for healthy adult imposed-slow-speed versus their chosen speed. The absence of *tKE* and *sKE* is notable for slow speed walking in healthy adults (Figure 2), with its absence producing a number of striking effects, including the following:

(1) Magnitude of TE oscillation (change in value across the swing phase) was reduced for slow speed to 0.04 J/kg m; versus chosen speed of 0.23 J/kg m.
(2) *KE* components for both thigh and shank (*tKE*, *sKE*) were significantly lower for healthy slow speed, so low as to be negligible (<0.01).
(3) *tPE* change in energy level from toe off to maximum hip flexion (Δ*tPE_subphase2_*) was diminished for slow speed (mean = 0.015 (±0.01) J/kg m) versus chosen speed (mean = 0.04 (±0.02) J/kg m; *p* = 0.001; Table 1).
(4) Absent thigh within-thigh energy conservation in early swing; that is, between*tPE* and *tKE* there was an absence of *tKE*, rendering no possibility of within-thigh energy conservation between *PE* and *KE* (Figure 3).
(5) Diminished energy transfer from the shank *KE* to the thigh (*PE*) in late swing, resulting from the absence of *sKE* oscillation (Figure 3).

### 3.2. Stroke Survivors

We observed two main compensatory strategies that were exhibited by the stroke survivors as follows: stepping strategy (n = 11); and circumduction strategy (n = 3). We conducted separate analyses for these two different gait compensatory strategies in stroke survivors.

#### 3.2.1. Stroke Step Strategy

##### Similarities in Limb Segment Mechanical Energies for Stroke Stepping Strategy Versus Slow Speed-Matched Controls

**Figure 4:**
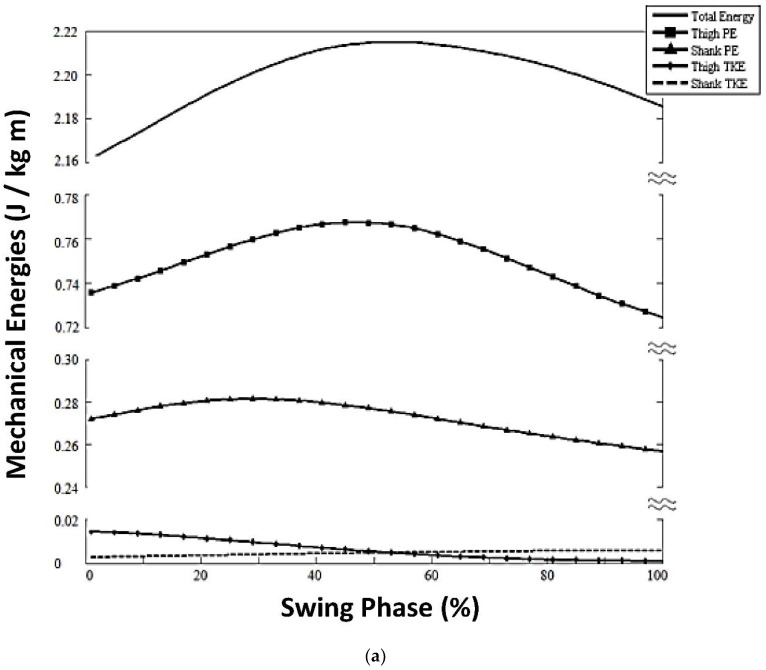

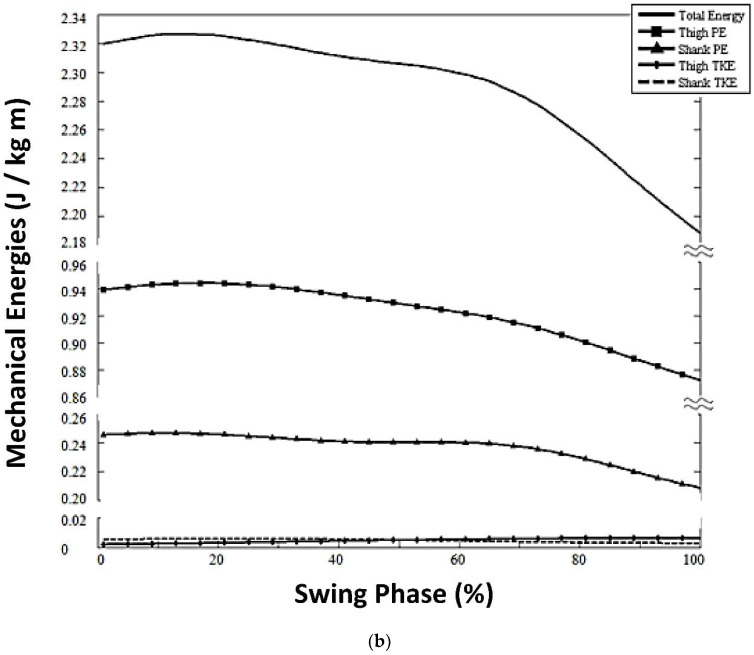
(**a**). Mechanical energies for stroke survivors using the stepping compensatory strategy. (**b**). Mechanical energies for stroke survivors using the stiff-legged circumducting compensatory strategy.

(1) *KE* components for both thigh and shank (*tKE*, *sKE*) were significantly different and so low as to be negligible (Figure 4).
(2) Timing of peak *tPE* (52%) in the swing phase (Figure 2; Panel B, Stepping Gait) as with the speed-matched controls (60% swing phase; Figure 2, Panel A, Slow Speed).

##### Differences in Limb Segment Mechanical Energies for Stroke Step Strategy Versus Slow Speed-Matched Controls

(1) Late TE peak at 51% of swing for stroke stepping strategy versus slow healthy adults’ TE peak at 25% of swing (comparing Figure 4a versus Figure 3, respectively).
(2) *sPE* peaked later in the swing phase at 33% (±17) of the gait cycle for stroke stepping strategy versus slow healthy adults at 15% (±7); *p* = 0.002, Figure 2, comparing Panel B, stepping strategy versus Panel A, healthy adult slow speed).
(3) Δ*sPE_Subphase1_* was significantly elevated, 0.01 (±0.01) J/kg m) for stroke stepping strategy versus slow healthy adults, 0.001 (±0.006) J/kg m; *p* = 0.036; Table 1). However, *sPE* had very little overall oscillation throughout the rest of the swing phase (Figure 4a); therefore, for the stroke stepping strategy, the general potential energy trends were not present as they were for slow healthy adults. That is, the normally decreasing *sPE* was not present at the time that *tPE* normally rose across mid-swing (comparing Figure 4a, Stroke Stepping Strategy versus Figure 3, Slow Healthy Adults).

#### 3.2.2. Stroke Circumduction Strategy

##### Similarities in Limb Segment Mechanical Energies for Stroke Circumduction Strategy Versus Slow Speed-Matched Healthy Adults

(1) *KE* profiles of both the thigh and the shank were near-zero (Figure 4b).

##### Differences in Limb Segment Mechanical Energies for Stroke Circumduction Strategy Versus Slow Speed-Matched Healthy Adults

(1) Earlier total energy (TE) peak and then a drastic decrease until heel strike (Figure 4b, solid line).
(2) Earlier peak thigh *PE* at 22% swing phase versus slow healthy adults with peak at 60% swing (*p* = 0.011, Figure 2).
(3) Greater peak thigh *PE* magnitude, 0.93 (±0.02) J/kg m), versus speed-matched controls with peak, 0.85 (±0.0.04) J/kg m; *p* = 0.014; Figure 4b).
(4) Diminished Δ*tPE_Subphase2_* magnitude, 0.01 (±0.011) J/kg m) versus slow healthy adults at 0.02 (±0.012; *p* = 0.014).
(5) Higher peak *sPE*, 0.23 (±0.02) J/kg m versus slow healthy adults, 0.21 (±0.011; *p* = 0.014; Table 1).

## 4. Discussion

The results of this study contribute to the literature in three ways, as follows: (1) quantification of mechanical energies (*KE* and *PE*) of the thigh and shank during the swing phase, at chosen speed for healthy adults, and discovery of underlying mechanical energy mechanisms contributing to optimizing physiological energy cost of normal chosen gait speed; (2) quantification of mechanical energies (*KE* and *PE*) of the thigh and shank during the swing phase, at imposed slow speed for healthy adults; comparison with chosen speed; and discovery of underlying mechanical energy mechanisms contributing to the greater physiological energy cost of normal slow speed gait; (3) quantification of mechanical energies (*KE* and *PE*) of the thigh and shank during the swing phase, for stroke survivors (for two different compensatory gait strategies); and discovery of underlying swing phase mechanical energy mechanisms contributing to the greater physiological energy cost of swing phase gait deficits after stroke.

### 4.1. Chosen Speed Walking: Limb Segment Mechanical Energy Characteristics of Healthy Adults

Our results provide the quantitative evidence explaining underlying mechanisms for two swing phase mechanical energy advantages for healthy adult chosen-speed walking, as follows: (1) Optimized total energy (TE) of thigh and shank during the swing phase limb movements; and (2) No knee flexor muscle activations required for early swing phase knee flexion. Both of these discoveries contribute to optimizing the physiological energy required from muscle activations.

#### 4.1.1. Optimized Total Mechanical Energy (TE) of Thigh and Shank during the Swing Phase Limb Movements

For the swing phase, the central nervous system (CNS) motor control problem is to move the ‘swing limb’ from behind the body (toe-touch position at pre-swing), forward to a position in front of the body (heel-strike), but simultaneously optimizing the physiological energy cost. Our results showed that conservation of mechanical energies occurs within and between thigh and shank, and is achieved through precise sequential peak times and through contrasting, complimenting changing levels of *KE* and *PE* within the thigh and between the thigh and shank (Figure 1).

Our results provide quantitative evidence to support that the CNS employs the following during the swing phase to achieve conservation of mechanical energies:

a. forward movement speed of the thigh and shank (*KE*) during chosen-speed walking, as they are carried forward by virtue of the forward sagittal plane pendular-like movement and also being attached to the torso, during its whole-body forward movement across the swing phase;
b. forces of gravity on thigh and shank during the vertical lifting and lowering movements (*PE*) of the thigh and shank during the swing phase;
c. the upper thigh segment attached to the body at the hip and the lower shank segment attached to the thigh at the knee; and thus, the shank is directly influenced by thigh biomechanics, as well as the mechanisms directly exerted on the shank;

To our knowledge, our results provide, for the first time, the quantitative evidence of thigh and shank values of *KE* and *PE* across the swing phase and how their precise interactions and conservations (between *KE* and *PE*) do optimize the physiological cost of the swing phase. Four key quantitative discoveries are listed in the Results Section 3.1.1. and which support these findings. These quantitative results are consistent with other types of data in case studies reported by others and suggestions and observations made by others [18,23].

For example, within the thigh, *PE* is at a relatively high value at toe-off, ready to contribute to *KE* when the swing phase begins. During early swing, as the thigh segment swings downward and forward, as *PE* decreases, it is then translated to *KE*, building *KE*, which then is utilized to ‘boost’ the thigh into its upward portion of the pendulum swing (hip flexion). At the point of maximum hip flexion of the swing phase, the *KE* has then been expended having assisted in the lifting of the thigh into maximum hip flexion; and *PE* has been increased, by virtue of the high thigh position (maximum hip flexion). This sequence of events was quantified and listed in ‘key finding’ #3 (Results Section 3.1.1.; Figure 1).

These mechanical energy mechanisms serve to minimize the required mechanical total energy (TE) of the thigh and shank and produce smooth changes in TE across the swing phase. In turn, these optimized mechanical energy requirements reduce the work required to move the limbs, reducing the physiological energy cost of limb movements during the swing phase.

#### 4.1.2. No Muscle Activations Required for Swing Phase Knee Flexion; Underlying Mechanism Quantified

The shank mechanical energies are complicated by the fact that the shank is attached to the thigh, which locates it anatomically as the lower segment of a double pendular-like system. Therefore, in addition to the shank’s own movements and interactions of *KE*/*PE* mechanical energies, the shank is also influenced by the thigh *KE* and *PE* energies. For example, as thigh *KE* is being expended to lift the thigh, the shank *KE* increases. This contrast occurs when the thigh is moving forward by virtue of its forward pendular-like trajectory in the sagittal plane and forward whole-body movement; as this occurs, the thigh ‘brings the shank along’ in a forward direction, increasing the shank *KE* (which is dependent upon forward speed of movement of the shank). The momentum of the upward pendular trajectory of the thigh also ‘brings the shank along’ in its upward trajectory, raising the shank and causing an increase in shank *PE* (based on the increasing height of the shank above ground). This phenomenon was quantified and listed in key discovery #4 (Results Section 3.1.1.). Since the shank is attached by the knee joint to the thigh, the structure of the knee joint constrains the direction of pendular movement of the shank; that is, knee joint flexion can occur in the sagittal plane, in the direction that is opposite to the direction of forward body movement. This structural fact allows the shank to take full advantage of the interactions of *KE* and *PE* from the thigh in allowing knee flexion to occur in response to increasing shank *KE* and *PE*. Evidently, this conservation of energy across thigh and shank and the anatomical structure of the limbs and joints are enough to flex the knee without the need for muscle activation [3,24,25].

We can note that in chosen speed walking for healthy adults, it is well-known that knee flexion movement during the early swing phase is accomplished with no muscle activations [24], but to our knowledge, to date, there is no available quantitative information explaining this phenomenon. Our results provide the quantitative evidence of the underlying mechanism that makes this possible. That is, our results showed that *KE* and *PE* interactions within the thigh and across the shank and thigh precisely utilize and expend mechanical energies in such a manner as to not only conserve mechanical energies, but also produce the knee flexion movement in the early swing phase, without the need for muscle activation of knee flexors in early swing.

### 4.2. Effect of Slow Speed Walking on Healthy Adults

During imposed slow speed walking (<0.4 m/s), the advantage of *KE* was lost. The limb segments were moved with extremely low velocity throughout the swing phase, resulting in near-zero magnitudes of *tKE* and *sKE*, and also causing lower magnitude of overall TE oscillation. Therefore, during slow walking for healthy adults, key advantages of normal energy transfers were not available due to the absence of normal magnitude of *tKE*. First, there was no longer optimal transfer of mechanical energies within the thigh segment (*KE*/*PE*), which normally would have occurred during 0–56% of swing; that is, at chosen speed, there was decreasing *tKE* and increasing *tPE* during that subphase of gait. In contrast, during slow walking there was an abnormally low range of *tPE* magnitude between toe off and maximum hip flexion at the slow speed for healthy adults (Δ*tPE_subphase2_*).

Second, for slow walking, there was no longer a transfer of mechanical energy between thigh and shank segments, which normally would have occurred at 0–35% of swing. Specifically, at chosen speed, there was decreasing *tKE*, increasing *sPE* and increasing *sKE*. At slow speed, there was an absence of the normal peaking of *tKE* at the end of the stance phase (beginning of the swing phase); therefore, other factors were required to flex the hip and lift the thigh against gravity, namely hip flexor muscle activity. In healthy adults, normal control of muscle activations is readily available for slow speed walking, with the well-known swing phase segments moving forward in the sagittal plane, but at a slower speed and with diminished joint movement excursions in some joints. These results may serve as a partial explanation as to the underlying mechanisms causing elevated work for healthy adults at imposed slow walking [1,2,21].

### 4.3. Limb Segment Mechanical Energy Characteristics of Stroke Survivors

For stroke survivors with gait deficits, slow walking speed is imposed by virtue of either or both balance impairment and limb joint discoordinated movements. Just as in healthy adults at slow gait speed, our results showed that stroke survivors walking at their chosen very slow speed had negligible *KE* in both thigh and shank. The result, as in healthy adults at slow speed, was the absence of conservation of mechanical energies (*KE*/*PE*) within the thigh and across the thigh and shank segments. In the absence of *KE* during the swing phase and in the absence of mechanisms of conservation of energy within the thigh and across the thigh and shank, stroke survivors were forced to employ some other means to advance the swing limb.

However, in contrast to healthy adults, the stroke survivors were not able to employ muscle activations for normal balance control and normal joint movement coordination that were exhibited during the slow speed gait of healthy adults. Rather, the stroke survivors exhibited compensatory strategies for the swing phase according to residual muscle function available to advance the swing limb. In our stroke survivor sample, there were two compensatory swing phase patterns employed: Stepping Strategy or Circumduction.

Those using the Stepping Strategy exhibited an abnormally elevated range of shank *PE* from toe off to maximum hip flexion. In healthy adults, there is a predictable sequence of knee flexion (35% of swing) followed by hip flexion (70% of swing; [3], Figure 1), which contributes to that same sequence of potential energies; that is, the first peak is *sPE* followed by the peak *tPE*. For the stroke survivors, however, hip and knee flexion were not coordinated in the normal, precise sequential manner; rather, hip and knee were flexed simultaneously with the effect that the hip was over-flexed, lifting the thigh higher than in normal adults at slow speed, in order for the limb to clear the floor. *PE* is a function of height from ground, and so this compensatory strategy resulted in abnormally elevated *sPE*. In the absence of *tKE* and *sKE*, there was no available mechanism to conserve mechanical energy and minimize physiological energy cost.

For those using the Circumduction Strategy, during the swing phase, the shank was carried first laterally and then forward by the circumducting thigh movement, with the knee fully extended throughout. In this manner, thigh *PE* peaked abnormally early, occurring at nearly the same time as shank *PE* (Figure 2). That phenomenon, as well as the negligible *KE*’s, prevented energy conservation within the thigh as well as energy transfer from shank to thigh in late swing.

Symptoms after stroke can include heightened muscle tone producing abnormal co-contractions at a given joint. For example, though knee joint flexion movement normally occurs during the swing phase, after stroke the knee extensors are abnormally activated, preventing knee flexion [26,27]. Abnormal knee co-contractions can result in a circumducted gait. For the stroke survivors exhibiting a circumducting compensatory strategy, the knee did not flex during the swing phase. Others reported abnormally high level of mechanical work needed for stroke subjects with ‘stiff-knee’ gait [28]. The circumduction strategy certainly had an adverse effect on the total energy profile (TE, Figure 4b), which showed a drastic rate of decrease in late swing. This high negative slope is indicative of inefficient mechanical energy relationships. Ultimately, the result yields an abnormally elevated level of mechanical work needed for the swing phase [19]. Thus, it is reasonable to consider that the inability to use efficient mechanical energies within and across thigh and shank during the swing phase has a bearing on the poor walking endurance reported by stroke survivors [1,2].

### 4.4. Contributions to the Field

In constructing gait training protocols after stroke, it is critical to target the underlying impairments and pathologies preventing the normal coordinated gait pattern. Our results indicate that the CNS normally can incorporate advantageous *KE* and *PE* energy conservation within the thigh and across thigh and shank that contribute to optimizing the energy cost of walking. In order to employ those mechanical advantages, normal muscle activations are required for joint movement and balance control. Abnormal muscle tone after stroke can interfere with the coordinated flexion and extension of hip and knee movements (26) that allow normal *KE* and *PE* energy conservation during the swing phase. In that case, treatment should be targeted to improve motor control of hip, knee, and ankle, flexion and extension movements required for sagittal plane, swing phase, coordinated movements. We can note that conservation of energy between *KE* and *PE* within the thigh and across the thigh and shank appears to accomplish the knee flexion movement in early swing without muscle activation of knee flexors for normal chosen speed. This supports a gait training intervention with greater attention to and resolution of the abnormal and interfering co-contractions that can occur at the hip and knee during swing the phase. The treatment goal, then, would be to restore motor control of both muscle activations and de-activations, so that the limb segments (thigh and shank) are released to move freely in response to normally available mechanical energies, and with a minimum optimal set of muscle activations. If balance impairment is present, abnormal and interfering extensor muscle activations can be active during the swing phase [29], which then can prevent normal hip and knee joint flexion required for the swing phase. Therefore, treatment should be targeted to improve balance control needed for double limb support, transfer of weight from trailing to forward limb, and dynamic single limb support, so that this potential source of abnormal co-contraction can be mitigated, as well.

We can provide one note of caution. Our results do not support simply forcing a ‘faster’ gait pattern because this could produce a faster pattern characterized by unsafe gait deficits, which would not necessarily resolve impairment in joint movement coordination or balance control.

### 4.5. Limitations

Small sample size is a limitation in this study, overall. Therefore, results should be considered with the standard cautions regarding generalization from small sample size to the population. A second limitation is that we focused our work on thigh and shank limb segments; though relatively small in value, future work could include the foot mechanical energies. A third limitation is that we focused on the swing phase; future work could include mechanical energies of across the entire gait cycle for thigh, shank, and foot.

## 5. Conclusions

### 5.1. Healthy Adults 

Healthy adults walking at chosen speed exhibited key mechanical advantages produced by the kinetic (*KE*) and potential (*PE*) energies of the thigh and shank, and conservation of mechanical energies (*KE* and *PE*) both within thigh and shank and across thigh and shank. These mechanical energy patterns during the swing phase serve to smooth the total energy pattern of the lower limb swing phase (TE), as well as drive swing phase knee flexion without the need for muscle activation of the knee flexors. These two discoveries at least partially explain how knee flexion movement is accomplished without muscle activation, as well as explaining the optimal energy cost of chosen gait speed for healthy adults.

At slow walking speed for healthy adults, there was negligible *KE* for thigh and shank, with the loss of conservation of mechanical energies. This resulted in the requirement of muscle activations to produce limb swing phase and forward advancement. This discovery provides at least one mechanism underlying the known higher physiological energy cost of slower walking versus chosen speed walking in healthy adults.

### 5.2. Stroke Survivors 

Stroke survivors walking at slow gait speed exhibit the same loss of thigh and shank *KE* as do healthy adults at slow gait speed. However, in contrast to healthy adult slow gait, there were greater requirements for muscle function in lifting the limb vertically, as reflected in higher *PE* values, as the swing limb was either over-flexed at the hip (Stepping Strategy) or swung laterally from the hip (Circumducing Strategy). Results of the study showed that these two compensatory gait strategies after stroke were characterized by disadvantages in mechanical energetics that were greater than those exhibited by healthy controls walking at slow speed, partially explaining the higher cost of slow walking in stroke survivors versus healthy adults. Implications for treatment include coordination training of lower limb movements and balance training; study results indicated the necessity to free the lower limb from abnormal co-contractions at a given joint so as to allow the lower limb to swing freely, according to its double-pendular structure and concomitant conservation of mechanical energies of the thigh and shank during the swing phase of gait.

## Figures and Tables

**Table 1:** Group comparisons of energy characteristics.

	A. Controls at Chosen vs. Slow Speed			B. Controls (Slow) vs. Stroke Step Strategy			C. Controls (Slow) vs. Stroke Circ Strategy		
	Controls	Controls	*p*-Value	Controls	Stroke	*p*-Value	Controls	Stroke	*p*-Value
	Chosen Speed	Slow Speed		Slow Speed	Step Strategy		Slow Speed	Circ Strategy	
*tPE_PeakAmplitude_* (J/kg-m)	0.85	0.85	0.856	0.85	0.86	0.468	0.85	0.93	0.014 *
	(±0.04)	(±0.04)		(±0.04)	(±0.05)		(±0.04)	(±0.02)	
*tPE_timing_*	51.00	60.00	0.007 *	60.00	51.62	0.173	60.00	22.00	0.011 *
(% swing)	(±4.70)	(±8.13)		(±8.13)	(±15.60)		(±8.13)	(±10.37)	
*sPE_PeakAmplitude_*	0.21	0.21	0.182	0.21	0.22	0.251	0.21	0.23	0.014 *
(J/kg-m)	(±0.01)	(±0.01)		(±0.01)	(±0.04)		(±0.01)	(±0.02)	
*sPE_timing_*	29.00	15.00	0.003 *	15.00	33.00	0.002 *	15.00	10.00	0.692
(% swing)	(±9.07)	(±7.30)		(±7.30)	(±17.00)		(±7.30)	(±7.658)	
Δ*sPE_subphase1_*	0.02	0.001	0.0001 *	0.001	0.01	0.036 *	0.001	0.00	0.811
(J/kg-m)	(±0.01)	(±0.005)		(±0.006)	(±0.01)		(±0.006)	(±0.003)	
Δ*tPE_subphase2_*	0.04	0.015	0.001 *	0.017	0.02	0.863	0.02	0.01	0.014 *
(J/kg-m)	(±0.02)	(±0.01)		(±0.012)	(±0.01)		(±0.012)	(±0.011)	
*tKE_PeakAmplitude_*	0.17	0.01 ^+^	0.0001 *	0.01 ^+^	0.01 **^+^**	--	0.01 ^+^	0.01 **^+^**	--
(J/kg-m)	(±0.06)	(±0.002)		(±0.002)	(±0.01)		(±0.002)	(±0.01)	
*tKE_timing_*	5.00	--	--	--	--	--	--	--	--
(% swing)	(±9.38)								
*sKE_PeakAmplitude_*	0.18	0.01	0.0001 *	0.01 ^+^	0.01 **^+^**	--	0.01 ^+^	0.01 **^+^**	--
(J/kg-m)	(±0.06)	(±0.002)		(±0.002)	(±0.01)		(±0.002)	(±0.01)	
*sKE_timing_*	27.00	--	--	--	--	--	--	--	--
(% swing)	(±6.74)								

* Significant difference between the two groups (*p* ≤ 0.05); ^+^ *KE* value was <0.08; and, therefore, by definition, considered to have a negligible effect. No further analyses were conducted.

## Data Availability

Data associated with this paper are provided in the manuscript.
